# An Autonomous Circadian Clock in the Inner Mouse Retina Regulated by Dopamine and GABA

**DOI:** 10.1371/journal.pbio.0060249

**Published:** 2008-10-14

**Authors:** Guo-Xiang Ruan, Gregg C Allen, Shin Yamazaki, Douglas G McMahon

**Affiliations:** Department of Biological Sciences, Vanderbilt University, Nashville, Tennessee, United States of America; University of Geneva, Switzerland

## Abstract

The influence of the mammalian retinal circadian clock on retinal physiology and function is widely recognized, yet the cellular elements and neural regulation of retinal circadian pacemaking remain unclear due to the challenge of long-term culture of adult mammalian retina and the lack of an ideal experimental measure of the retinal circadian clock. In the current study, we developed a protocol for long-term culture of intact mouse retinas, which allows retinal circadian rhythms to be monitored in real time as luminescence rhythms from a PERIOD2::LUCIFERASE (PER2::LUC) clock gene reporter. With this in vitro assay, we studied the characteristics and location within the retina of circadian PER2::LUC rhythms, the influence of major retinal neurotransmitters, and the resetting of the retinal circadian clock by light. Retinal PER2::LUC rhythms were routinely measured from whole-mount retinal explants for 10 d and for up to 30 d. Imaging of vertical retinal slices demonstrated that the rhythmic luminescence signals were concentrated in the inner nuclear layer. Interruption of cell communication via the major neurotransmitter systems of photoreceptors and ganglion cells (melatonin and glutamate) and the inner nuclear layer (dopamine, acetylcholine, GABA, glycine, and glutamate) did not disrupt generation of retinal circadian PER2::LUC rhythms, nor did interruption of intercellular communication through sodium-dependent action potentials or connexin 36 (cx36)-containing gap junctions, indicating that PER2::LUC rhythms generation in the inner nuclear layer is likely cell autonomous. However, dopamine, acting through D1 receptors, and GABA, acting through membrane hyperpolarization and casein kinase, set the phase and amplitude of retinal PER2::LUC rhythms, respectively. Light pulses reset the phase of the in vitro retinal oscillator and dopamine D1 receptor antagonists attenuated these phase shifts. Thus, dopamine and GABA act at the molecular level of PER proteins to play key roles in the organization of the retinal circadian clock.

## Author Summary

The circadian clock in the mammalian retina regulates many retinal functions, and its output modulates the central circadian clock in the brain. Details about the cellular location and neural regulation of the mammalian retinal circadian clock remain unclear, however, largely due to the difficulty of maintaining long-term culture of adult mammalian retina and the lack of an ideal experimental measure of the retinal clock. We have circumvented these limitations by developing a protocol for long-term culture of intact mouse retinas to monitor circadian rhythms of clock gene expression in real time. Using this protocol, we have localized expression of molecular retinal circadian rhythms to the inner nuclear layer. We find molecular retinal rhythms generation is independent of many forms of signaling from photoreceptors and ganglion cells, or major forms of neural communication within the inner nuclear layer, and have characterized light-induced resetting of the retinal clock. Retinal dopamine and GABA, although not necessary for the generation of molecular retinal rhythms, were revealed to regulate the phase and amplitude of retinal molecular rhythms, respectively, with dopamine participating in light-induced resetting. Our data indicate that dopamine and GABA play prominent roles in the organization of the retinal circadian clock.

## Abstract 3

Long-term culture of mouse retinas reveals a circadian clock in the inner retina that can be reset by light and is regulated by the neurotransmitters dopamine and GABA.

## Introduction

The vertebrate retina is both a sensory organ and an endogenous circadian clock. As the locus of visual phototransduction, the retina initiates many organismal responses to light, and as a circadian clock, the retina expresses many physiological or functional circadian rhythms, including photoreceptor disc shedding [1–4], visual sensitivity [5–8], rod–cone balance[9,10], electroretinogram (ERG) b-wave amplitude [11–13], extracellular pH [14], melatonin release [15–18], dopamine synthesis [19,20], gamma-aminobutyric acid (GABA) turnover rate and release [21], PKC level [22], intraocular pressure [23,24], and gene expression [13]. In mammals, the retina is the sole site for circadian phototransduction, and its output modifies the rhythmicity, period, and developmental organization of the central biological clock, the suprachiasmatic nucleus (SCN) of the hypothalamus [25–27]. In addition, the mammalian retinal clock and its outputs influence trophic processes in the eye, including the susceptibility of photoreceptors to degeneration from light damage [28], photoreceptor survival in animal models of retinal degeneration [29], and the degree of refractive errors in primate models of myopia [30]. Despite the widespread influence of the mammalian retinal circadian clock on retinal, visual, and circadian function, the cellular elements and mechanisms comprising the retinal pacemaker remain to be elucidated.

The mammalian retina contains all the major neurotransmitter systems, and to a large extent, retinal function is mediated by neurotransmission. Glutamate is the prominent excitatory neurotransmitter of the neuron types of vertical pathways through the retina, including photoreceptors, bipolar, and ganglion cells [31,32]. The other major excitatory neurotransmitter, acetylcholine (ACh), is produced by a mirror-symmetric pair of amacrine cells [33]. The inhibitory neurotransmitter GABA occurs in many different subtypes of amacrine cells and in one or more subtypes of horizontal cells [34]. The inhibitory neurotransmitter glycine is present in most of the small-field types of amacrine cells [35]. Besides these four fast neurotransmitters, there are two globally modulatory neurotransmitters, melatonin and dopamine, which are mutually inhibitory neurochemical outputs of the retinal clock and are rhythmically released by photoreceptors and dopaminergic amacrine cells, respectively [19,36,37]. Melatonin and dopamine rhythms have served as the principal experimental measures of the retinal circadian clock, and previous models of retinal circadian organization have postulated that communication through these two transmitters is critical for overall retinal rhythmicity [15,19,20,38,39]. However, these neurochemical assays of clock output have left open questions of whether dopamine or melatonin modulates the retinal molecular clock mechanism itself and what roles they may play in the organization of retinal clockworks.

Circadian clocks in mammalian tissues generate molecular circadian rhythms through coupled transcription/translation feedback loops in which the positive gene elements *Clock* and *Bmal1* interact with the negative gene elements *Period* (*Per*) *1* and *2*, and *Cryptochrome* (*Cry*) *1* and *2,* and in which casein kinases, CKIɛ and CKIδ, set circadian period by phosphorylating PER proteins to regulate their degradation and nuclear localization [38,40]. Circadian rhythms of *Per1* expression have been reported in the rat and mouse retina [41,42]. We have previously shown that in photoreceptor-degenerate mouse retinas, *Per1*, *Per2*, *Cry1*, *Cry2*, and *Bmal1* mRNA levels exhibit statistically significant variations over a 24-h sampling period under constant darkness conditions [43]. Moreover, in a recent microarray study, 277 genes representing a wide range of functions were found to show a circadian rhythm of expression in the mouse retina in constant darkness [13]. However, it is unclear from these in vivo studies whether circadian rhythms of retinal gene expression are self-sustained or driven by the master SCN clock.

In the current study, we developed an in vitro retinal explant culture protocol to monitor circadian rhythms in clock gene expression in intact retinas of adult mice, which harbor a knockin PERIOD2::LUCIFERASE (PER2::LUC) fusion protein as a real-time reporter of circadian gene dynamics [44]. Using this protocol, we studied the properties and location of retinal PER2::LUC rhythms in vitro, the influence of major retinal neurotransmitters, the roles of sodium-dependent action potentials and connexin 36 (cx36)-containing gap junctions in retinal PER2::LUC rhythms, and light-induced phase resetting of retinal PER2::LUC rhythms. Our findings support a model of retinal circadian organization in which rhythm generation occurs in the inner nuclear layer independently of major forms of neural communication, but in which dopamine and GABA play critical roles in setting the phase and amplitude of the retinal circadian clock.

## Results

### PER2::LUC Rhythms from Intact Mouse Retinas

**Figure 1:**
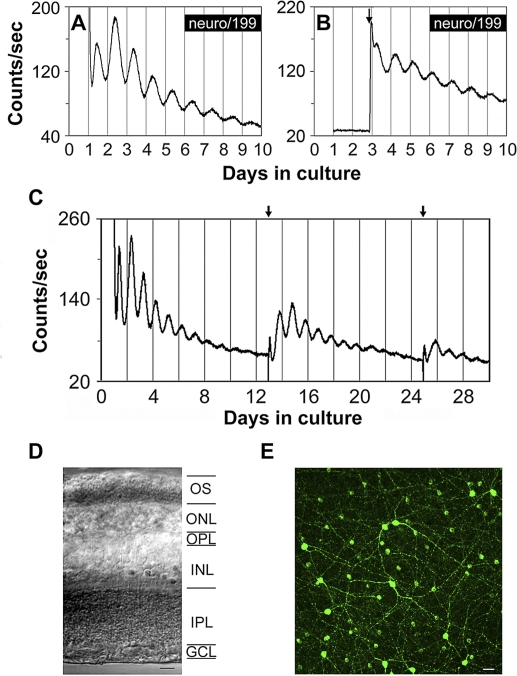
Bioluminescence Rhythms from *mPer2^Luc^* Mouse Retinal Explants (A) A representative PER2::LUC bioluminescence trace recorded from a B6C3 *mPer2^Luc^* mouse retinal explant. (B) A representative PER2::LUC bioluminescence trace recorded from a mouse retinal explant in which luciferin substrate was absent from the medium in the first two cycles. Arrow indicates addition of luciferin. (C) Long-term culture of an intact mouse retinal explant showing persistent circadian rhythms in PER2::LUC expression. Arrows indicate times of media changes. (D) Representative DIC image of vertical retinal sections. Retinal explants were cultured for 9 d in vitro (DIV), and vertical retinal slices were cut with a tissue slicer at 150 μm. GCL, ganglion cell layer; INL, inner nuclear layer; IPL, inner plexiform layer; ONL, outer nuclear layer; OPL, outer plexiform layer; OS, photoreceptor outer segments. Scale bar represents 20 μm. (E) Flat-mount view showing tyrosine hydroxylase immunoreactivity in retinal explants that were cultured for 9 DIV. The immunoreactive cells with relatively large somata and two to three thick primary processes that arise from the cell body are dopaminergic amacrine cells, whereas the immunoreactive cells with relatively small cell body and very few processes are type 2 catecholamine amacrine cells. Scale bar represents 20 μm.

To study the retinal circadian clock in the intact retina, *mPer2^Luc^* mice congenic on the C57BL/6J background, in which melatonin levels are greatly reduced due to point mutations in synthetic enzymes for melatonin [19,45], were crossed to C3H *rd1*/*rd1* mice to obtain F1 hybrid B6C3 mice. These mice have one wild-type allele at the melatonin and *rd1* loci, synthesize and secrete normal levels of melatonin [46], and do not undergo photoreceptor degeneration. When retinal explants from B6C3 F1 mice were cultured under standard conditions (medium 199 or DMEM, air, 37 °C), they exhibited only low-amplitude PER2::LUC oscillations (Figure S1). However, when retinal explants were first cultured in neurobasal medium in 5% CO_2_ at 37 °C for 24 h in vitro and subsequently transferred to medium 199 as before, PER2::LUC expression was robustly rhythmic for numerous circadian cycles (Figure 1A). Peak-to-trough amplitude typically increased on the second cycle in medium 199, after which the amplitude gradually decreased over 9–10 d (Figure 1A). The first peak of PER2::LUC expression occurred at hour 14.81 ± 0.29 of projected Zeitgeber time (ZT; i.e., 2.81 h after the time of lights off in the mouse colony; mean ± standard error of the mean [SEM]; *n* = 11). This was essentially identical to the phase of PER2::LUC rhythms in photoreceptor-degenerate retinas [43], and phase delayed by 2 h relative to the peak times of SCN PER2::LUC rhythms [44]. Retinal *Per2* mRNA abundance is rhythmic in vivo, as sampled at 4-h intervals by quantitative reverse transcriptase (RT)-PCR (Figure S2), indicating that the luminescence signals recorded in vitro faithfully reflect in vivo rhythmic processes. The phase of the in vivo *Per2* and of the in vitro PER2::LUC rhythms were similar, suggesting that the culture procedure itself does not greatly affect the phase of retinal molecular oscillations. However, the limited resolution of the in vivo data (4-h intervals) prevented a more precise definition of the expected delay between *Per2* transcription and PER2::LUC luminescence. As with the cultured SCN [47], a media change partially restored the amplitude of luminescence rhythms (Figure 1C). With media changes every 10 d, retinal rhythms could be readily sustained for a month or more.

To control for whether bioluminescence light emission produced by the PER2::LUC fusion protein influenced retinal rhythms, we performed assays in which luciferin substrate was absent from the medium, and thus, there was no retinal light emission for the first two circadian cycles in vitro. Luciferin (0.1 mM) was then added to the medium of experimental explants, and an equal volume of vehicle was added to the medium of control explants at the beginning of the rising phase of the third cycle recorded in the LumiCycle. The delayed addition of luciferin revealed rhythms of PER2::LUC luminescence in the experimental explants that were similar in amplitude and peak time to the ongoing rhythms of control explants (*n* = 4; Figure 1B), indicating that bioluminescence light emitted by the PER2::LUC reporter in these retinas did not influence retinal PER2::LUC rhythms in our in vitro assay.

Histological examination of vertical slices from cultured retinas confirmed that all retinal layers were intact in these preparations, including photoreceptor cell bodies and outer segments (Figure 1D). In addition, immunocytochemistry with an antibody to tyrosine hydroxylase (TH), the rate-limiting enzyme of dopamine synthesis, confirmed that cultured retinal explants retained a normal complement of dopamine neurons (Figure 1E). Further immunocytochemical analysis revealed that all the major retinal cell types, including cone photoreceptors and horizontal, bipolar, amacrine, and ganglion cells, were present and morphologically intact in cultured retinal explants (Figure S3).

### Circadian Oscillation of PER2::LUC Bioluminescence in the Inner Nuclear Layer

**Figure 2:**
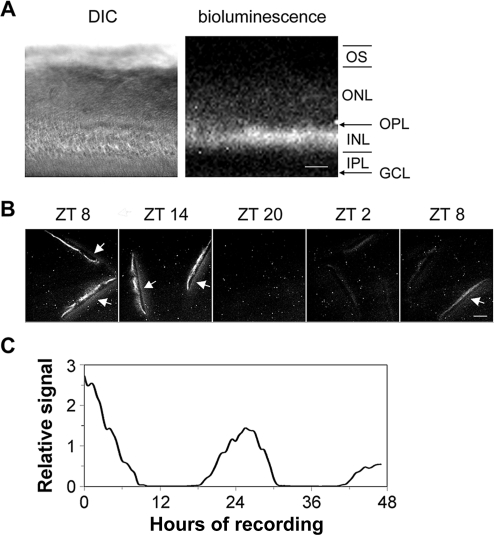
Circadian Oscillation of PER2::LUC Bioluminescence in the Inner Nuclear Layer (A) DIC image (left panel) and luminescence image (right panel) of a vertical retinal slice. Retinal slices were prepared around ZT 4, incubated in CO_2_ incubator for 1 d, and then imaged. Note that bioluminescence signals were primarily located in the inner nuclear layer. GCL, ganglion cell layer; INL, inner nuclear layer; IPL, inner plexiform layer; ONL, outer nuclear layer; OPL, outer plexiform layer; OS, photoreceptor outer segments. Scale bar represents 50 μm. (B) PER2::LUC bioluminescence in the inner nuclear layer of cultured vertical retinal slices showed a circadian variation with a peak at projected ZT 8–14. Shown are representative images acquired by 30-min exposures at 6-h intervals. Arrows point to membrane filters that became visible when signals in the inner nuclear layer were high. Scale bar represents 100 μm. (C) Continuous recording of PER2::LUC bioluminescence in a representative cultured vertical retinal slice. In this experiment, retinal slices were prepared around ZT 4, incubated in CO_2_ incubator for 2 d, and then images (30-min exposure) were continuously collected for 47 h, starting at projected ZT 9.

Multiple approaches, including in situ hybridization, immunocytochemistry, and single-cell reverse-transcriptase PCR (RT-PCR), have established that the *Per2* clock gene is expressed, with varying levels and frequencies, in all the major subtypes of retinal neurons [13,43,48]. Thus, all retinal cell subtypes could potentially contribute to the tissue-level PER2::LUC luminescence rhythms recorded in our assays. To localize the PER2::LUC signal within the retina, we imaged vertical retinal slices for both bright-field and luminescence with a cooled charge-coupled device (CCD) camera. The bioluminescence signal was concentrated in the mid-retina, in the inner nuclear layer that contains the nuclei of the horizontal, bipolar, and amacrine cells, with low levels of luminescence detected in the photoreceptor and the ganglion cell layers (Figure 2A). To confirm that inner retinal PER2::LUC is rhythmic, populations of retinal slices maintained in constant culture conditions were imaged at 6-h intervals (*n* = 2 independent runs; Figure 2B). PER2::LUC bioluminescence in the inner nuclear layer exhibited a circadian variation with a peak at projected ZT 8–14, close to the peak time observed in whole retinal explant preparations. In addition, individual retinal slices subject to time-lapse imaging for 48 h showed similar circadian rhythms in PER2::LUC bioluminescence emanating from the inner nuclear layer (*n* = 3 independent runs; Figure 2C). Similar inner nuclear layer localization of PER2::LUC expression was observed in retinas from C57BL/6J mice as well (unpublished data). This indicates that the primary source of rhythmic PER2::LUC luminescence signals in our retinal rhythms assay is the inner nuclear layer, with minor contributions from the other retinal layers.

### PER2::LUC Rhythms in the Inner Nuclear Layer Are Not Dependent upon Communication from Photoreceptors or Ganglion Cells

Although the inner nuclear layer was the primary source of rhythmic PER2::LUC signals in our vertical retinal slice imaging, signaling from photoreceptors or ganglion cells might play an important role in generating or sustaining these rhythms. To test this possibility, we examined the influence of the main forms of cell communication from photoreceptors (melatonin and glutamate) and ganglion cells (glutamate, sodium-dependent action potentials, and cx36-containing gap junctions) on retinal PER2::LUC rhythms.

**Figure 3:**
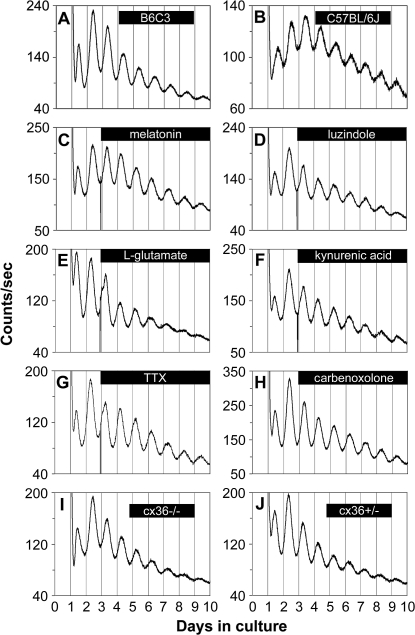
Retinal PER2::LUC Rhythms Do Not Require Communication via Melatonin, Glutamate, Sodium-Dependent Action Potentials or cx36-Containing Gap Junctions (A) *mPer2^Luc^* retinal explants cultured from B6C3 mice exhibited sustained circadian rhythms in PER2::LUC expression. (B) *mPer2^Luc^* retinal explants cultured from C57BL/6J mice also showed robust circadian oscillation of PER2::LUC expression. (C–H) Circadian PER2::LUC expression rhythms persisted upon continuous application of: (C) melatonin (10 nM); (D) the melatonin MT1 receptor antagonist luzindole (5 μM); (E) l-glutamate (1 mM); (F) the broad-spectrum glutamate receptor antagonist kynurenic acid (0.5 mM); (G) the voltage-gated sodium channel blocker TTX (1 μM); and (H) the gap junction block carbenoxolone (100 μM). (I and J) Retinal explants cultured from cx36^−/−^ mice (I) and cx36^+/−^ mice (J) did not show differences in the damping rate of PER2::LUC rhythms. Bars indicate the duration of treatment.

To determine whether melatonin signaling is required to maintain retinal rhythmicity in clock gene expression, we assayed luminescence rhythms from retinal explants derived from *mPer2^Luc^* C57BL/6J mice, in which melatonin synthesis is genetically blunted [19,45]. Circadian rhythms of PER2::LUC expression in C57BL/6J retinas were similar in their sustained nature to those of B6C3 F1 retinas (*n* = 6 each; Figure 3A and 3B), which synthesize and secrete normal levels of melatonin [46]. Similarly, B6C3 retinas, treated with continuous application of 10 nM melatonin or continuous blockade of melatonin MT1 receptors with 5 μM luzindole, also exhibited robust circadian gene expression rhythms (*n* = 6 each; Figure 3C and 3D). Taken together, these results indicate that the mouse retina requires neither normal levels of melatonin nor melatonin rhythmicity to maintain PER2::LUC rhythms.

Glutamate is an excitatory neurotransmitter used by photoreceptors, ganglion, bipolar, and amacrine cells. To examine the influence of glutamate on retinal PER2::LUC rhythms, we either activated its receptors with l-glutamate (1 mM; *n* = 4; Figure 3E), or blocked its receptors with kynurenic acid (0.5 mM; *n* = 4; Figure 3F), a broad-spectrum glutamate receptor antagonist. Neither of these treatments exhibited marked effects on the amplitude or period of retinal PER2::LUC rhythms (*n* = 3 each), indicating that glutamatergic neurotransmission is not required for maintenance of retinal PER2::LUC rhythms.

In the mammalian retina, ganglion cells and dopaminergic amacrine cells require sodium-dependent action potentials for evoked neurotransmitter release and subsequent chemical communication [49]. In addition, in the SCN pacemaker, sodium-dependent action potentials are critical for molecular rhythms generation in individual neurons and for cellular synchronization [50]. Thus, we tested the role of sodium-dependent action potentials in the retinal circadian pacemaker with the sodium channel blocker tetrodotoxin (TTX), previously shown to damp tissue and cellular gene expression rhythms in in vitro SCN cultures [50,51]. Retinal PER2::LUC rhythms persisted undiminished upon continuous application of 1 μM TTX, and there was no significant difference in e-fold damping rate between TTX treatment and vehicle (3.92 ± 0.22 d vs. 3.82 ± 0.34 d; *p* > 0.05; *n* = 5 each; Student's *t*-test; Figure 3G). This concentration of TTX was confirmed to block spontaneous action potentials in ganglion cells and in dopaminergic neurons (unpublished data). Therefore, sodium-dependent action potentials are not required for retinal PER2::LUC rhythms generation.

Ganglion cells form extensive electrically coupled networks via gap junctions. To test for the role of gap junctions, we applied 100 μM carbenoxolone, a general gap junction blocker, to *mPer2^Luc^* retinal explants. Carbenoxolone did not influence the amplitude or damping rate of retinal PER2::LUC rhythms (damping rate = 3.54 ± 0.18 days; *n* = 5; Figure 3H). In addition, we examined the role of the gap junction channel protein, cx36 in retinal PER2::LUC rhythms. Cx36 is widely distributed in retinal gap junctions, is essential for transmission of rod-mediated visual signals in the mammalian retina [52], and comprises most of the junctions between alpha ganglion and amacrine cells [53]. Interneuronal communication via cx36 gap junctions also promotes synchronous electrical activity in SCN neurons and coherent circadian behavioral rhythms [54]. To test whether cx36-mediated electrical coupling plays a role in the retinal biological clock, we crossed *mPer2^Luc^* mice with cx36^−/−^ mice [55] to produce cx36 knockout *mPer2^Luc^* mice. PER2::LUC rhythms in retinas from both cx36^−/−^ and cx36^+/−^ mice were similar to mice wild type at the cx36 locus, and there was no significant difference in the damping rate between cx36^−/−^ mice and cx36^+/−^ mice (2.62 ± 0.12 d vs. 2.74 ± 0.33 d; *p* > 0.05; *n* = 8 each; Figure 3I and 3J), suggesting that neuronal synchrony was not degraded by cx36 knockout. Taken together, these results suggest that communication through gap junctions is also not required for retinal PER2::LUC rhythms generation or for circadian neural synchrony in the mouse retina.

### Retinal PER2::LUC Rhythms Are Not Dependent upon Communication within the Inner Nuclear Layer

The wide distribution of PER2::LUC expression across the inner nuclear layer suggests the possibility that multiple inner nuclear layer neuron types, including horizontal, bipolar, and amacrine cells, could be sources for the rhythmic PER2::LUC signals. Rhythms generation could be a network property driven by a specific population of pacemaker neurons located in the inner nuclear layer, which establish PER2::LUC rhythmicity by neurotransmission. To test this possibility, we examined the role of the principal neurotransmitters of the inner nuclear layer: dopamine, acetylcholine, GABA, and glycine.

**Figure 4:**
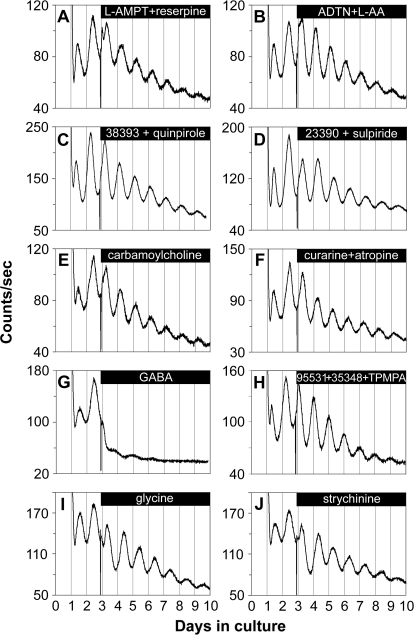
Retinal PER2::LUC Rhythms Are Not Dependent on Communication via Dopamine, Acetylcholine, GABA, or Glycine (A–F) Retinal circadian PER2::LUC expression rhythms persisted upon continuous application of: (A) the TH inhibitor L-AMPT (100 μM) along with the vesicular dopamine uptake inhibitor reserpine (10 μM); (B) the dopamine receptor agonist ADTN (100 μM), in the presence of L-AA (100 μM); (C) the dopamine D1 receptor agonist SKF-38393 (50 μM) along with the D2/D4 receptor agonist quinpirole hydrochloride (50 μM); (D) the dopamine D1 receptor antagonist SCH-23390 (50 μM) along with the D2 receptor antagonist sulpiride (50 μM); (E) the nonselective cholinergic agonist carbamoylcholine chloride (100 μM); and (F) the nicotinic acetylcholine receptor antagonist (+)-tubocurarine (100 μM) along with the muscarinic acetylcholine receptor antagonist atropine (100 μM). (G) GABA (1 mM) greatly suppressed the amplitude of retinal PER2::LUC rhythms. (H) Retinal PER2::LUC rhythms persisted during blockade of GABA receptors with the GABA_A_ receptor antagonist SR-95531 (40 μM) along with the GABA_B_ receptor antagonist CGP-35348 (100 μM) and the GABA_C_ receptor antagonist TPMPA (100 μM). (I and J) Retinal PER2::LUC rhythms persisted upon continuous application of: (I) glycine (3 mM); and (J) the glycine receptor antagonist strychnine hydrochloride (50 μM). Bars indicate the duration of treatment.

We first examined whether dopamine is required for retinal PER2::LUC rhythms generation. PER2::LUC rhythms persisted during depletion of retinal dopamine by 100 μM α-methyl-l-tyrosine (L-AMPT) and 10 μM reserpine, an inhibitor of dopamine synthesis and of vesicular dopamine uptake, respectively (*n* = 4; Figure 4A). Dopamine depletion at 48 h posttreatment was confirmed with high-performance liquid chromatography (HPLC). Whereas control retinas contained 0.93 ± 0.08 ng/mg protein dopamine, L-AMPT–treated retinas contained 0.50 ± 0.06 ng/mg protein, and L-AMPT– and reserpine-treated retinas contained undetectable levels of dopamine (*n* = 4 each). In addition, circadian PER2::LUC expression rhythms persisted during continuous application of the long-lasting dopamine agonist (±)-2-amino-6,7-dihydroxy-1,2,3,4-tetrahydronaphthalene (ADTN; 100 μM; in vehicle containing 100 μM l-ascorbic acid [L-AA, an antioxidant]; *n* = 4; Figure 4B). However, ADTN produced perturbations in the waveform during the first few cycles of treatment and indeed changed the phase of retinal PER2::LUC rhythms (further analyzed below). Retinal PER2::LUC rhythms also persisted during continuous application of the D1 receptor agonist SKF-38393 and the D2/D4 receptor agonist quinpirole hydrochloride (50 μM each; *n* = 4; Figure 4C); or continuous blockade of dopamine receptors with the D1 receptor antagonist SCH-23390 and the D2/D4 receptor antagonist sulpiride (50 μM each; *n* = 4; Figure 4D). These data, along with our above-mentioned results demonstrating that retinal PER2::LUC rhythms persisted in the presence of TTX, form convergent lines of evidence suggesting that neither dopaminergic transmission nor its rhythmicity is necessary for ongoing retinal PER2::LUC circadian rhythms generation.

The excitatory neurotransmitter ACh is employed by specialized circuits and produced by a mirror-symmetric pair of amacrine cells. Neither activation of ACh receptors with the nonselective cholinergic agonist carbamoylcholine chloride (100 μM; *n* = 4; Figure 4E), nor blockade of ACh receptors with a variety of antagonists including the nicotinic acetylcholine receptor antagonist (+)-tubocurarine (100 μM) alone (unpublished data; *n* = 4), the muscarinic ACh receptor antagonist atropine (100 μM) alone (unpublished data; *n* = 4), or (+)-tubocurarine and atropine together (*n* = 4; Figure 4F), had a noticeable effect on the amplitude or period of retinal PER2::LUC rhythms.

The inhibitory neurotransmitter GABA is synthesized and released by horizontal cells and several subtypes of amacrine cells including dopaminergic amacrine cells. Activation of GABA receptors with 1 mM GABA substantially suppressed the amplitude of retinal PER2::LUC rhythms (*n* = 4; Figure 4G). However, retinal PER2::LUC rhythms persisted during blockade of GABA receptors with the GABA_A_ receptor antagonist SR-95531 (40 μM) along with the GABA_B_ receptor antagonist CGP-35348 (100 μM) and the GABA_C_ receptor antagonist 1, 2, 5, 6-tetrahydropyridine-4-yl methyl phosphinic acid (TPMPA; 100 μM; *n* = 4; Figure 4H), indicating that GABAergic transmission is not required for retinal PER2::LUC rhythms generation. Manipulation of another inhibitory neurotransmitter glycine (3 mM; *n* = 4; Figure 4I) or the glycine receptor antagonist strychnine (50 μM; *n* = 4; Figure 4J) did not exhibit marked effects on the phase, amplitude, or period of retinal PER2::LUC rhythms, although the baseline of PER2::LUC rhythms was modestly reduced.

### Dopamine Sets the Phase of Retinal PER2::LUC Rhythms

**Figure 5:**
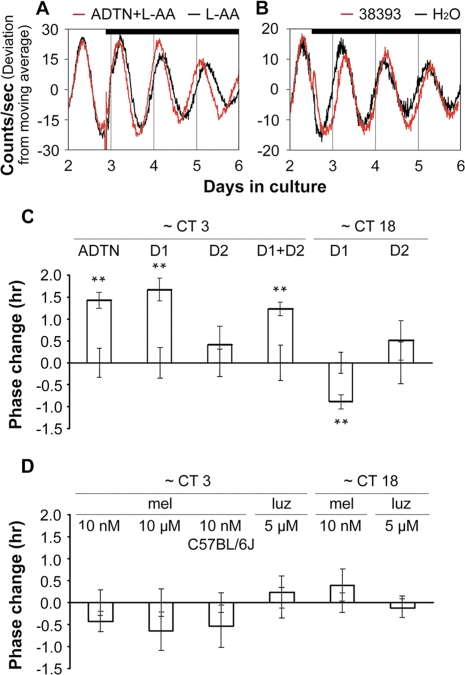
Dopamine Shifts the Phase of Retinal PER2::LUC Rhythms (A) Application of ADTN (100 μM) along with L-AA (100 μM) beginning at CT 3 advanced PER2::LUC rhythms compared to L-AA (100 μM) application alone. (B) Application of the D1 receptor agonist SKF 38393 (50 μM) beginning at CT 18 delayed PER2::LUC rhythms compared to vehicle (H_2_O). Data shown in (A) and (B) were baseline corrected by calculating a 24-h moving average of the raw data, and then the deviation from the moving average was plotted as a function of days in culture. Bars indicate the duration of treatment. (C) Phase change following application of dopamine agonists. Briefly, the peak times (as determined by ClockLab software) on the third cycle (after treatment) were subtracted from the peak times on the second cycle (before treatment) for both drug and vehicle, and then used to calculate the phase change of drug versus vehicle controls. Bars show drug phase changes (means ± SEM), error bars from *x* axis show ±SEM for vehicle controls in each group. Drug concentrations were as follows: ADTN, 100 μM; the D1 agonist SKF 38393, 50 μM; and the D2 agonist quinpirole, 50 μM. Double asterisks (**) indicate *p* < 0.01, Student's *t*-test; *n* = at least 4 for each drug treatment and for vehicle controls. (D) Lack of phase change following application of melatonin reagents. Phase change was calculated as in (C). Drug concentrations are indicated above columns. Vehicles used for 10 nM melatonin (mel), 10 μM melatonin, and 5 μM luzindole (luz) were 1 μl of 0.01% ethanol, 1 μl of 10% ethanol, and 1 μl of DMSO, respectively; *n* = at least 4 for each drug treatment and for vehicle controls. No significant effects were observed (*p* > 0.05 for all).

Manipulation of dopaminergic neurotransmission produced perturbations in the waveform of retinal PER2::LUC rhythms at the onset of treatments with dopaminergic agonists, but did not damp or disrupt rhythms generation (Figure 4A–4D). To test the effects of dopamine on retinal phase, we applied dopaminergic reagents in a step protocol beginning at different circadian times during the second cycle, using the peak time as a phase reference point of approximately circadian time (CT) 15. We then compared the transient change in period (phase shift) of PER2::LUC rhythms in reagent-treated explants relative to those of vehicle-treated explants on the cycle ensuing initiation of treatment (see Figure 5C legend for detail). Application of the nonselective dopamine receptor agonist ADTN (100 μM; in vehicle containing 100 μM L-AA), or the D1 dopamine agonist SKF 38393 (50 μM) beginning in the early retinal subjective day (CT 3), phase-advanced retinal rhythms by approximately 1.5 h (Figure 5A and 5C). In contrast, application of the D2/D4 receptor agonist quinpirole (50 μM) at CT 3, did not reset retinal phase (Figure 5C), whereas coapplication of the two agonists together produced phase advances similar in amplitude to D1 agonist alone (Figure 5C). Application of D1 agonist, beginning at CT 18, induced phase delays of approximately 1 h (Figure 5B and 5C), whereas application of the D2 agonist at that phase did not affect retinal phase (Figure 5C). Phase advances and delays induced by dopamine agonists were stable and persisted for multiple cycles. Although dopaminergic agonists were applied as a bolus and thus were potentially active for multiple circadian cycles, the periods of molecular rhythms of treated explants were only altered relative to controls for two cycles following administration of dopaminergic reagents and then stabilized, indicating that these reagents likely have a limited effective half-life of about 48 h in our culture system. Acute pulse application experiments, performed in an attempt to define more precisely the phase dependence of the resetting action of dopamine, did not yield useful data due to artifactual phase shifts induced by the repeated media changes themselves.

Manipulation of melatonin transmission did not have any noticeable effects on the waveform of retinal PER2::LUC rhythms (Figure 3C and 3D) and did not significantly affect the phase of retinal molecular rhythms. Neither application of melatonin (10 nM), nor the melatonin receptor antagonist luzindole (5 μM) significantly altered the phase of retinal PER2::LUC rhythms (Figure 5D). Melatonin and luzindole were each applied beginning at two different phases of the retinal rhythm, at approximately CT 3 or CT 18, but none of these treatments resulted in a statistically significant change in retinal phase (Figure 5D). In addition, application of a higher dose of melatonin (10 μM) to B6C3 retinas, or application of 10 nM melatonin to C57BL/6J retinas also failed to alter retinal phase (Figure 5D).

### Light Resetting of the Phase of Retinal PER2::LUC Rhythms Is Mediated In Part Through Dopamine D1 Receptors

**Figure 6:**
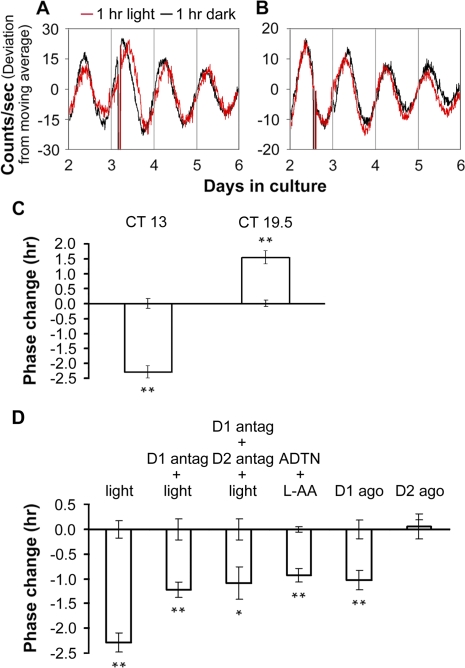
Light Resetting of the Phase of Retinal PER2::LUC Rhythms Is Mediated In Part through Dopamine D1 Receptors (A and B) Phase changes of retinal PER2::LUC rhythms induced by 1-h light pulses beginning at CT 13 (A) or CT 19.5 (B). Red traces show rhythms from explants that received light; black traces are from paired controls that were handled similarly, but did not receive light. (C) Average data of light-induced phase shifts. Error bars from *x*-axis show ±SEM for controls in which retinal explant cultures were taken out from the LumiCycle and kept in darkness at 37 °C for 1 h. (D) Phase changes of retinal PER2::LUC rhythms. Retinal explant cultures were subjected to or treated with: column 1, 1-h light pulses (a reproduction from [C]); column 2, pretreatment with the D1 receptor antagonist SCH-23390 (D1 antag; 50 μM) for 15 min followed by 1-h light pulses; column 3, pretreatment with SCH-23390 and the D2 antagonist sulpiride (D2 antag; 50 μM) together for 15 min followed by 1-h light pulses; column 4, the nonselective dopamine receptor agonist ADTN (100 μM; in vehicle containing 100 μM L-AA); column 5, the D1 agonist SKF 38393 (D1 ago; 50 μM); and column 6,the D2 agonist quinpirole (D2 ago; 50 μM). The 1-h light pulses or dopaminergic treatments were initiated at CT 13. Phase change was calculated as in Figure 5. A single asterisk (*) indicates *p* < 0.05; double asterisks (**) indicate *p* < 0.01; Student's *t*-test; *n* = at least 3 for each treatment and for controls.

To test the effects of light pulses on the phase of retinal PER2::LUC rhythms, we prepared retinal explant cultures under infrared illumination to preserve photoreceptor function and subjected them to 1-h pulses of white light (500 lux) at different circadian times during the second cycle in vitro. Light pulses beginning in the early retinal subjective night (CT 13) phase delayed retinal PER2::LUC rhythms by approximately 2.3 h, whereas light pulses beginning in the late retinal subjective night (CT 19.5) induced phase advances of approximately 1.5 h (Figure 6A–6C). To determine whether dopamine acting through D1 receptors mediates light resetting of retinal PER2::LUC rhythms, we applied the D1 receptor antagonist SCH-23390 (50 μM) beginning 15 min before starting 1-h light pulses at CT 13. Light-induced phase delays were significantly attenuated in the presence of SCH-23390 compared to light alone (1.2 h, *p* < 0.01, Figure 6D). Pretreating retinal explants with the D1 antagonist SCH-23390 and the D2 receptor antagonist sulpiride (50 μM) together prior to light pulse resulted in phase delays similar in amplitude to D1 antagonist pretreatment alone (Figure 6D). Application of the nonselective dopamine receptor agonist ADTN (100 μM; in vehicle containing 100 μM L-AA) or the D1 agonist SKF 38393 (50 μM) beginning at CT 13 in the absence of light exposure, induced phase delays of approximately 1 h (Figure 6D), which were significantly lower in amplitude than the phase delays produced by 1-h light pulses (*p* < 0.01). Application of the D2 agonist quinpirole at CT 13 did not reset retinal phase (Figure 6D).

### GABA Negatively Regulates the Amplitude of Retinal PER2::LUC Rhythms

**Figure 7:**
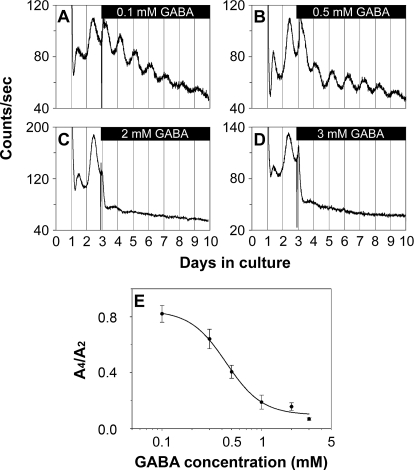
GABA Sets the Amplitude of Retinal PER2::LUC Rhythms in a Dose-Dependent Manner (A–D) Representative PER2::LUC bioluminescence traces recorded from cultured *mPer2^Luc^* retinal explants that received different doses of GABA treatment. Bars indicate the duration of GABA treatment. (E) Dose-dependent inhibitory action of GABA on the amplitude of retinal PER2::LUC rhythms. The ratio of the peak-to-trough amplitude of the fourth cycle to that of the second cycle (A_4_/A_2_) was plotted as a function of the external GABA concentration. Data are represented as mean ± SEM (*n* = at least 3 explants per point).

In Figure 4G, we found that 1 mM GABA greatly suppressed the amplitude of retinal PER2::LUC rhythms. We further tested different concentrations of GABA on our retinal explant cultures. Application of 0.1 mM GABA did not obviously alter PER2::LUC rhythms compared with vehicle (Figure 7A); however, at a higher concentration (0.5 mM), GABA acutely suppressed the level of PER2::LUC bioluminescence signals and greatly reduced the peak-to-trough amplitude of PER2::LUC oscillations on subsequent circadian cycles (Figure 7B). At 2 mM or 3 mM, GABA further inhibited PER2::LUC luminescence levels and resulted in rapid damping of PER2::LUC rhythms (Figure 7C and 7D). Media changes that removed GABA restored the damped oscillations (see below). To further characterize the inhibitory action of GABA on retinal rhythms, we calculated the ratio of the peak-to-trough amplitude of the fourth circadian cycle (second cycle after treatment) to the amplitude of the second cycle (pretreatment control cycle, A_4_/A_2_). This ratio was plotted as a function of GABA concentration (Figure 7E). The A_4_/A_2_ for 0.1 mM GABA was 0.82 ± 0.06 (*n* = 5), which was similar to that for vehicle treatment (0.75 ± 0.08; *n* = 5). As GABA concentration was increased, A_4_/A_2_ gradually decreased, and at 3 mM GABA, most explants lacked clear rhythms, and the few cycles that could be measured were of very low amplitude (0.07 ± 0.01; *n* = 7), indicating that GABA inhibits PER2::LUC rhythmic amplitude in a dose-dependent manner. However, GABA did not significantly change the period of retinal PER2::LUC rhythms compared with vehicle (τ = 23.80 ± 0.16 h for 0.5 mM GABA, *n* = 6; τ = 24.33±0.22 h for 1 mM GABA, *n* = 7; and τ = 24.05 ± 0.09 h for vehicle, *n* = 5; *p* > 0.05 for both comparisons to vehicle).

To test whether the inhibitory action of GABA is specific to the retina, or common to other neural clock tissues, we applied 1 mM or 3 mM GABA to mouse *mPer2^Luc^* SCN explants. GABA did not significantly change the A_4_/A_2_ of SCN PER2::LUC rhythms compared to vehicle (0.91 ± 0.08 for 1 mM GABA; 0.86 ± 0.07 for 3 mM GABA; 0.84 ± 0.11 for vehicle treatment; *p* > 0.05 for both; *n* = 3 each; Figure S4); neither did GABA have significant effect on the period of SCN PER2::LUC rhythms (τ = 23.56 ± 0.26 h, 23.96 ± 0.32 h, and 23.63 ± 0.23 h for 1 mM GABA, 3 mM GABA, and vehicle, respectively; *p* > 0.05 for both comparisons to vehicle; *n* = 3 each). Therefore, GABA-induced inhibition of ensemble PER2::LUC rhythms is retina specific.

### GABA_A_ and GABA_C_ Receptors Mediate the Effects of GABA on Retinal PER2::LUC Rhythms

**Figure 8:**
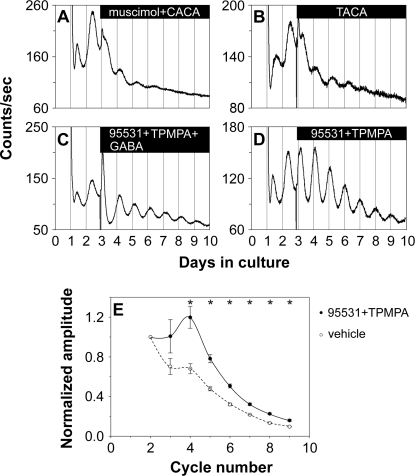
GABA Acts through GABA_A_ and GABA_C_ Receptors (A) Coapplication of the GABA_A_ receptor agonist muscimol (200 μM) and the GABA_C_ receptor agonist CACA (50 μM) to retinal explants mimicked the inhibitory action of 1 mM GABA on retinal PER2::LUC rhythms. (B) The GABA_A_ and GABA_C_ receptor agonist TACA (80 μM) inhibited PER2::LUC expression rhythms in a way similar to 1 mM GABA. (C) The GABA_A_ receptor antagonist SR-95531 (40 μM) and the GABA_C_ receptor antagonist TPMPA (100 μM) greatly attenuated the action of GABA when they were coapplied with 1 mM GABA. (D) Coapplication of SR-95531 (40 μM) and TPMPA (100 μM) increased the amplitude of retinal PER2::LUC rhythms. Bars in (A–D) indicate the duration of treatment. (E) Blockade of GABA_A_ and GABA_C_ receptors with 40 μM SR-95531 and 100 μM TPMPA (solid line, filled circles) significantly increased the amplitude of retinal PER2::LUC rhythms compared with vehicle-treated explants (dashed line, open circles). Mean peak-to-trough amplitudes (±SEM, *n* = 5; normalized to the amplitude of the second circadian cycle) of retinal PER2::LUC rhythms were plotted as a function of PER2::LUC cycle number in the LumiCycle. A single asterisk (*) indicates *p* < 0.007, post hoc *t*-test with Bonferroni-corrected α = 0.007.

Next, we characterized the receptors responsible for the inhibitory action of GABA. GABA receptors are classified as ionotropic, chloride-conducting GABA_A_ and GABA_C_ receptors, or as metabotropic GABA_B_ receptor. All three types of GABA receptors are present in the mammalian retina [31]. When the GABA_A_ receptor agonist muscimol (200 μM) or the GABA_C_ agonist *cis*-4-aminocrotonic acid (CACA; 50 μM) was applied individually to the retinal explants, the amplitude of retinal PER2::LUC rhythms was significantly reduced compared to that of vehicle-treated samples (see Table 1 for A_4_/A_2_ ratios of our GABA pharmacological treatments; Figure S5A and S5C). However, the amplitude of PER2::LUC rhythms was largely unaffected by the GABA_B_ agonist baclofen (200 μM; Figure S5B). When muscimol and CACA were applied together, they mimicked the inhibition of 1 mM GABA on retinal PER2::LUC rhythms (Figure 8A). Baclofen coapplied with either muscimol or CACA, or baclofen coapplied with both muscimol and CACA, did not enhance the inhibition of the GABA_A_ and GABA_C_ agonists (Figure S5D–S5F). *trans*-4-Aminocrotonic acid (TACA; 80 μM), an agonist for both GABA_A_ and GABA_C_ receptors, inhibited luminescence rhythms to a degree similar to 1 mM GABA (Figure 8B). These results indicate that activation of both GABA_A_ and GABA_C_ receptors is necessary to mimic fully the action of GABA on retinal rhythms, with GABA_B_ receptors playing no apparent role, either alone or in combination with the other receptors.

**Table 1:**
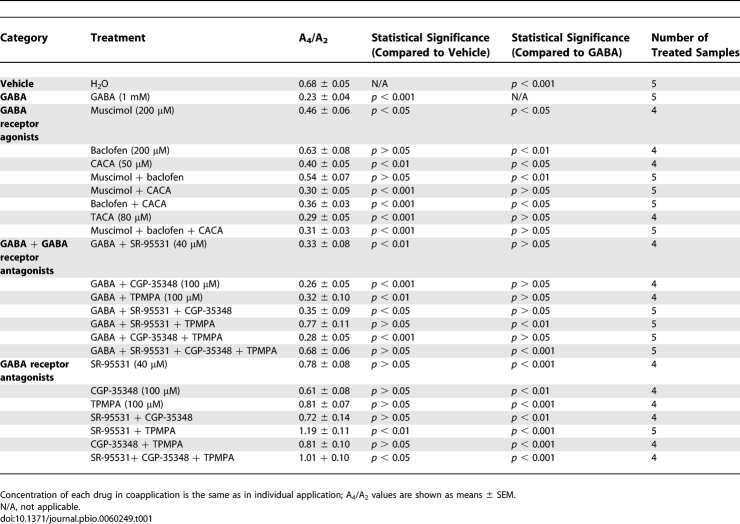
Summary of GABA Pharmacological Data

We further examined whether specific antagonists could block the action of GABA. The inhibitory effect of 1 mM GABA persisted when it was applied in the presence of the GABA_A_ receptor antagonist SR-95531 (40 μM) alone, the GABA_B_ receptor antagonist CGP-35348 (100 μM) alone, and the GABA_C_ receptor antagonist TPMPA (100 μM) alone (Figure S6A–S6C). However, the effect of 1 mM GABA was greatly attenuated by coapplication of SR-95531 and TPMPA (Figure 8C). Coapplication of either SR-95531 with CGP-35348, or TPMPA with CGP-35348 did not block the action of 1 mM GABA (Figure S6D and S6E). CGP-35348 also did not significantly enhance the blocking ability of SR-95531 and TPMPA when it was coapplied with both antagonists (Figure S6F). These results indicate that blockade of both GABA_A_ and GABA_C_ receptors is necessary to attenuate GABA inhibition of retinal rhythms with, again, no dependence on GABA_B_ receptors.

To assess the role of endogenous GABA in retinal rhythms generation, we next applied various GABA receptor antagonists to cultured *mPer2^Luc^* retinal explants. When GABA_A_ or GABA_C_ antagonists SR-95531 (40 μM) and TPMPA (100 μM) were applied individually, each modestly increased the peak-to-trough amplitude of luminescence rhythms (Figure S7A and S7C), whereas application of the GABA_B_ antagonist CGP-35348 (100 μM) alone did not change the amplitude (Figure S7B). However, antagonism of both GABA_A_ and GABA_C_ receptors with SR-95531 and TPMPA significantly increased the peak-to-trough amplitude of retinal ensemble PER2::LUC luminescence rhythms compared with vehicle (Figure 8D and 8E), indicating that endogenous GABA indeed suppresses the amplitude of retinal PER2::LUC rhythms. Again, CGP-35348 did not enhance the effect of SR-95531 and TPMPA (Figures 4H, S7D, and S7E). Taken together, these pharmacological studies indicate that endogenous retinal GABA reduces PER2::LUC signals and the amplitude of molecular retinal circadian rhythms through activation of both GABA_A_ and GABA_C_ receptors.

### Prolonged Application of GABA Stops the Retinal Clock

**Figure 9:**
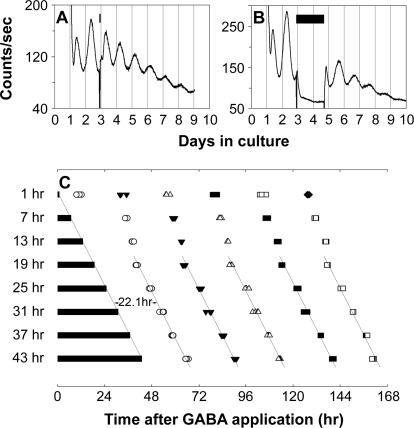
Prolonged Application of GABA Stops the Retinal Clock (A and B) Representative retinal explant cultures that received 1 h (A) or 43 h (B) of 3 mM GABA treatment. GABA treatment was started at the beginning of the third circadian cycle. Bars indicate the duration of GABA treatment. Treatment was terminated by fresh media change. (C) Shown are PER2::LUC expression peaks following GABA washout. Four retinal explants were sampled for each duration of GABA treatment. Bars indicate the duration of GABA treatment. Open circles, filled triangles, open triangles, filled squares, open squares, and filled diamonds indicate first, second, third, fourth, fifth, and sixth peak times, respectively, following GABA washout. Time 0 corresponds to the start of GABA treatment. In the samples with 19–43 h of GABA application, the first peaks appeared ca. 22 h after media change, and the reinitiated rhythms were phase locked to the termination of the GABA pulse, indicating that prolonged application of high dose of GABA stops retinal ensemble PER2::LUC rhythms.

To further test whether GABAergic suppression of PER2::LUC luminescence rhythms results from an effect on the core function of the retinal clock, we tested whether prolonged application of GABA can halt the molecular oscillation of the retinal clock. GABA (added to a final concentration of 3 mM in 1 μl of water vehicle) was applied to retinal cultures for different durations (1, 7, 13, 19, 25, 31, 37, and 43 h), starting at the beginning of the third cycle in vitro (Figure 9A and 9B). We hypothesized that if retinal rhythms generation was stopped by prolonged GABA application, and restored upon washout, then the time of washout should predict the subsequent phase of restored rhythms. In contrast, if the observed suppression of PER2::LUC luminescence rhythms during GABA application did not have an effect on the core clock, but merely on luminescence output, then the phase of restored rhythms should be predicted by the projected phase of the baseline rhythm obtained prior to treatment. Figure 9C shows the peak times of retinal PER2::LUC rhythms following GABA treatment and washout at the specified time intervals. For retinas exposed to GABA for 19 h or greater, the first peak of the restored luminescence rhythm always appeared ca. 22 h following GABA washout, with subsequent peaks occurring approximately at 24-h intervals. The trend lines of peak times show that the phase of restored rhythms is indeed predicted by the time of washout, but not by projected continuation of previous rhythms. In a control experiment, 1 μl of water vehicle was applied to retinal cultures for 37 h and then the medium was changed, as in the GABA application experiments. In this case, the first peak of the ongoing PER2::LUC rhythms occurred ca. 14 h after the media change, not ca. 22 h after, as when GABA was washed out (*n* = 3). Thus, GABA affects the retinal clock, not just its output, and its action in this experiment is consistent with having the ability to stop the molecular clock mechanism at a certain phase.

### GABA Acts Posttranscriptionally through Membrane Polarization and Casein Kinase

**Figure 10:**
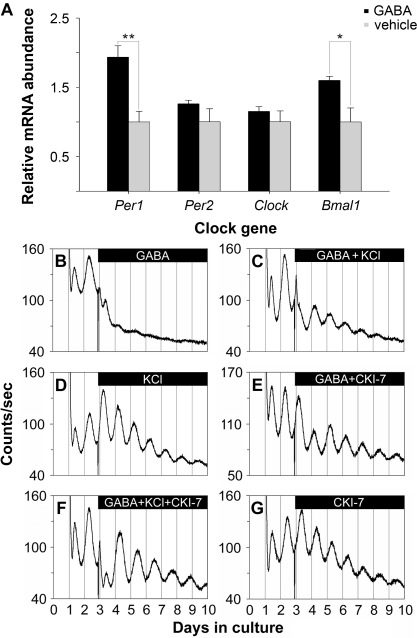
GABA Acts Posttranscriptionally through Membrane Polarization and Casein Kinase (A) Shown are relative mRNA abundance of *Per1*, *Per2*, *Clock*, and *Bmal1* in retinal explants 8 h after treatment with 3 mM GABA (black) or vehicle (grey) started at the beginning of the third circadian cycle. Clock gene mRNA levels were normalized to the mRNA levels of GAPDH. The average mRNA levels of vehicle-treated samples were set to 1.0. Data are presented as means ± SEM (*n* = 4). GABA did not significantly change the transcript levels of *Per2* and *Clock*, while significantly increased the transcript levels of *Per1* and *Bmal1*. A single asterisk (*)indicates *p* < 0.05; double asterisks (**) indicate *p* < 0.01; Student's *t*-test. (B) GABA (1 mM) greatly suppressed retinal PER2::LUC rhythms. (C) Depolarization with elevated K^+^ (4 mM) partially blocked the inhibitory effect of GABA (1 mM) on retinal PER2::LUC rhythms. (D) Prolonged application of KCl (4 mM) modestly increased rhythmic PER2::LUC amplitudes. (E) Inhibition of casein kinase activity with CKI-7 (50 μM) partially rescued the inhibitory action of 1 mM GABA on retinal PER2::LUC rhythms. (F) KCl (4 mM) and CKI-7 (50 μM) greatly rescued the inhibitory action of GABA (1 mM) on retinal PER2::LUC rhythms when they were co-applied with GABA. (G) When CKI-7 (50 μM) was applied alone, it lengthened the period of retinal PER2::LUC rhythms. Bars in (B–G) indicate the duration of treatment.

To determine the mechanisms by which GABA suppressed retinal PER2::LUC expression and rhythmicity, we first quantified *Per1*, *Per2*, *Clock*, and *Bmal1* mRNA levels 8 h after 3 mM GABA treatment using quantitative real-time PCR. Interestingly, GABA did not change the mRNA levels of *Per2* and *Clock*, but did increase the mRNA levels of *Per1* and *Bmal1* (Figure 10A). Therefore, the substantial reduction in PER2::LUC expression upon GABA treatment is likely due to posttranscriptional regulation.

The finding that GABA acts on the retinal PER2::LUC rhythms through ionotropic, Cl^−^-conducting receptors suggested that GABAergic modulation of the clock could be mediated by membrane hyperpolarization or, in the case of prolonged GABA application, by alteration of membrane ionic gradients leading to tonic depolarization [56]. Depolarization with elevated K^+^ media (4 mM) during 1 mM GABA application partially restored the rhythmic amplitude of PER2::LUC expression (A_4_/A_2_ = 0.70 ± 0.04 for vehicle, 0.20 ± 0.05 for GABA, and 0.45 ± 0.07 for 4 mM K^+^ + GABA; *n* = 5 each; Figure 10B and 10C), indicating that GABA acts in part through membrane hyperpolarization. Prolonged application of high K^+^ alone modestly increased the amplitude of PER2::LUC rhythms (A_4_/A_2_ = 0.90 ± 0.10; *n* = 5; Figure 10D), indicating that the inhibitory effect of GABA on retinal PER2::LUC rhythms is not due to tonic depolarization. The effect of GABA is not mediated through the simple cessation of neuronal spiking due to membrane hyperpolarization, because blocking neuronal spiking with TTX (1 μM) did not affect retinal PER2::LUC rhythms (A_4_/A_2_ = 0.67 ± 0.09; *n* = 5; Figure 3G).

Depolarization with elevated K^+^ media in the presence of GABA only partially rescued the amplitude of retinal PER2::LUC rhythms, indicating that other mechanisms contribute to the inhibitory action of GABA. The epsilon and delta isoforms of CKI are important regulators of PER protein stability that phosphorylate PER2 and target it for ubiquitin-mediated proteasomal degradation [57]. In addition, casein kinases associate with GABA_A_ and GABA_C_ receptor subunits [58,59]. To test whether casein kinases are involved in the inhibitory effect of GABA on PER2::LUC levels and retinal rhythms, we applied the casein kinase inhibitor CKI-7 (50 μM) along with 1 mM GABA to *mPer2^Luc^* retinal explant cultures. CKI-7 partially rescued rhythmic amplitude in the presence of GABA (A_4_/A_2_ = 0.35 ± 0.03 for CKI-7 + GABA vs. 0.20 ± 0.05 for GABA; *p* < 0.05; *n* = 5 each; Figure 10E). When KCl (4 mM) and CKI-7 (50 μM) were coapplied with 1 mM GABA, the rescue of rhythmic PER2::LUC amplitude was complete (A_4_/A_2_ = 0.93 ± 0.15; *n* = 4; Figure 10F). When applied alone, CKI-7 did not significantly change the amplitude of PER2::LUC rhythms; however, it substantially lengthened retinal free-running period from 24.05 ± 0.09 h (*n* = 5) to 25.77 ± 0.38 h (*n* = 6; *p* < 0.005; Figure 10G). Taken together, these results suggest that GABA acts, in part, by stimulating casein kinase to suppress PER2 levels.

## Discussion

In the present study, we developed a retinal explant culture protocol that enables the assay of circadian clock gene expression rhythms in real time from isolated, intact *mPer2^Luc^* mouse retinas. Using this protocol, we characterized retinal PER2::LUC expression rhythms, localized their source within the retinal layers, examined the influence of multiple retinal neurotransmitters, as well as sodium-dependent action potentials and cx36-containing gap junctions, and characterized light resetting of retinal oscillations. We found that isolated mouse retinas exhibited sustained, endogenous circadian rhythms in PER2::LUC expression localized primarily to the inner nuclear layer of the mouse retina, the generation of which was independent of many forms of signaling from photoreceptors and ganglion cells, or major forms of neural communication within the inner nuclear layer. Endogenous retinal dopamine and GABA, although not necessary for retinal PER2::LUC rhythms generation, were found to play key roles in regulating the retinal circadian clock: mediating light resetting of rhythmic phase and influencing the amplitude of retinal PER2::LUC rhythms, respectively.

### Culture of Mouse Retina

Information regarding the mechanisms of the mammalian retinal clock has been limited by the challenge of long-term culture of the mammalian retina and the interpretational caveats imposed by using melatonin or dopamine neurochemical output rhythms as proxies for observing the clock mechanism. The protocol we have developed here, a modification of the explant culture method for SCN luminescence recording [60], allows continuous readout of the rhythmic abundance of a putative molecular component of the circadian clock mechanism, PER2 protein, as bioluminescence intensity emitted by isolated, intact mouse retinal explants. Within our preparations, all layers of the retina were anatomically intact, and the retinas exhibited robust circadian rhythms of PER2::LUC expression as well as functional light responses in the form of phase resetting. A key empirical finding in developing this culture protocol was that incubation of the retina in bicarbonate-buffered medium for the first 24 h in vitro greatly enhanced the amplitude and sustainability of retinal PER2::LUC rhythms. The precise mechanism by which the transition through bicarbonate-based medium preserves retinal rhythmicity is unknown; however, bicarbonate balance has wide-ranging effects on retinal physiology, and increased bicarbonate concentration has previously been shown to increase the amplitude of circadian rhythms in photoreceptor disc shedding in amphibian retinas [61].

### An Inner Nuclear Layer Retinal Clock

Imaging of PER2::LUC luminescence signals from retinal vertical slice cultures of both B6C3 and C57BL/6J mice showed that PER2::LUC rhythms were predominantly localized to the inner nuclear layer of the retina, which contains the nuclei of retinal horizontal, bipolar, and amacrine cells. The pattern of PER2::LUC expression in the current study is consistent with previous single-cell RT-PCR and in situ hybridization studies demonstrating concentration of clock gene transcripts in the inner nuclear layer of the mouse [42,43,62], and immunocytochemistry studies showing predominant localization of PER1, PER2, and CLOCK proteins in the inner nuclear layer as well [13,42]. Localization of rhythmic PER2::LUC expression to the inner nuclear layer is consistent with the persistence of PER2::LUC rhythms in photoreceptor-degenerate mouse retinas [43], and with the persistence of *Per2* rhythms in photoreceptor-degenerate RCS retinas [63]. The fact that disruption of communication through glutamate, sodium-dependent action potentials, and cx36-containing gap junctions did not disrupt retinal rhythms further suggests that communication through ganglion cells is not necessary. The wide distribution of PER2::LUC expression across the inner nuclear layer suggests that multiple inner nuclear layer cell types are sources for the rhythmic PER2::LUC signal and are likely participants in the inner retinal clock mechanism. Cell-type–specific mapping of coordinate expression of the core circadian clock genes has demonstrated that significant proportions of horizontal, rod bipolar, and dopaminergic amacrine neurons of the inner nuclear layer express all six core clock genes and thus are candidate cell types for self-sustained circadian rhythms generation [43].

Although, our results suggest that the inner nuclear layer of the intact mouse retina contains a circadian clock, our study does not preclude the potential for circadian rhythms generation in the photoreceptor or ganglion cell layers. Indeed, Tosini et al. [64] have recently reported gene rhythms from photoreceptor layers isolated from rat retinas that carry a transcriptional transgenic reporter for *Per1*, rather than the knockin fusion protein reporter for PER2 used here.

### Cell Communication and Rhythms Generation

Our findings support cell-autonomous rhythms generation in the inner nuclear layer clock rather than rhythms generation as a network property dependent upon cell communication. None of the major neurotransmitter systems in photoreceptors (melatonin and glutamate), ganglion cells (glutamate), or the inner nuclear layer (dopamine, acetylcholine, GABA, glycine, and glutamate) was required to support generation of retinal circadian PER2::LUC rhythms, nor was intercellular communication through sodium-dependent action potentials and cx36-containing gap junctions, although dopamine and GABA exhibited important effects on retinal PER2::LUC rhythms. A question that arises for interpretation of these negative findings regarding cellular communication is the duration over which pharmacological treatments were effective in vitro. For GABA agonists and antagonists, it is clear that they are effective for at least 7 d (Figures 4, 7, and 8). For many other target transmitter systems, we have included genetic or chemical alternative tests that mitigate against this concern. For example, melatonin has been depleted genetically, as well as manipulated pharmacologically; dopamine has been depleted chemically, as well as manipulated pharmacologically; and gap junctional communication through the principal neural connexin, cx36, has been lesioned genetically to complement pharmacological disruption of electrical synapses. All of these alternative manipulations gave negative results consistent with the pharmacology. Blockade of sodium-dependent action potentials with TTX has previously been shown to be effective in disrupting circadian rhythms in the SCN cultured under similar conditions for several days [50,65]. In addition, TTX blockade of sodium-dependent action potentials itself would have significantly reduced the spike-mediated release of GABA, glycine, glutamate, acetylcholine, and dopamine by amacrine and ganglion cells of the inner retina, reinforcing the negative findings with the pharmacological blockers specific to each of these transmitter systems. Thus, we cannot exclude all possibility that these transmitter systems contribute to circadian rhythms generation in the retina; however, the consistent results we have obtained with overlapping types of treatments suggest that these negative results are robust. We also cannot rule out the possibility that multiple transmitter systems play redundant roles, or the possibility that some untested neurochemical signals are critical, but our data do exclude previous models of retinal circadian organization in which a limited population of cells using one of the modulatory neurotransmitters acts as a pacemaker for all retinal neurons, such as the 500 dopaminergic amacrine cells in each retina driving all other retinal oscillator cells through dopamine secretion, or the photoreceptors driving all other retinal oscillators through melatonin release [38].

### Regulation of the Inner Retinal Circadian Clock by Light and Dopamine

It is well established that dopamine and melatonin are important neurochemical messengers of the retinal circadian clock that act at multiple sites within the retinal circuitry to shape retinal function into “day” and “night” states [38,39,66,67]. Our results distinguish the roles of these neurotransmitters in retinal circadian organization, indicating that whereas dopamine has the additional role of regulating the phase of the core clock mechanism, melatonin's role is primarily that of an output messenger of the clock. Our finding that PER2::LUC rhythms persist in C57BL/6J mouse retinas with genetically blunted melatonin production and following pharmacological manipulation of melatonin signaling is consistent with previous studies showing that melatonin is not required for circadian photoreceptor disk shedding in constant darkness [4], and that photoreceptors, the primary source of retinal melatonin, are not required for retinal PER2::LUC rhythms generation [43]. In addition, in the present study, neither manipulation of melatonin nor blockade of MT1 receptors significantly altered the phase of retinal PER2::LUC rhythms. Taken together, these results suggest that melatonin is an output messenger of the clock, which can influence dopamine release and other aspects of retinal physiology, but which does not directly affect or feed back onto the molecular clock mechanism.

In contrast, dopamine is a key regulator of the endogenous retinal clock mechanism. Stimulation of dopamine D1 receptors reset the phase of retinal PER2::LUC rhythms, whereas blockade of dopamine D1 receptors reduced the amplitude of phase delays induced by light pulses given in the early subjective night. These effects on circadian phase demonstrate that retinal dopamine influences the core clock mechanism of mammalian retinas. Our findings suggest D1 dopamine receptors are key to the action of dopamine on the retinal circadian pacemaker, whereas D2 receptors have been suggested to play a role in light-induced *Per* gene induction by dopamine [68]. Both studies suggest that dopamine targets retinal cell types in the inner nuclear layer clock to reset the retinal circadian oscillator. The potential targets of phase resetting include multiple cell types that are the primary sites of D1 receptor expression in the inner nuclear layer: horizontal, AII amacrine, and bipolar cells [69]. The lack of phase setting of the inner retinal oscillator by the D2/D4 agonist quinpirole and melatonin suggests that photoreceptors and melatonin do not participate in the dopaminergic resetting process, as D4 receptors are expressed predominantly in retinal photoreceptors where they influence melatonin synthesis [69]. The D1-dependent resetting mechanism, which targets neurons of the inner nuclear layer, is distinct from dopamine-mediated light resetting of amphibian retinal melatonin rhythms, which involves D2-like receptors on photoreceptor cells [70,71].

Our demonstration of light resetting of in vitro retinal PER2::LUC rhythms is consistent with previous findings that the melatonin secretion rhythms of the mouse retina could be synchronized to light cycles in vitro [17] . All retinal photoreceptive cell types—rods, cones, and melanopsin ganglion cells—are anatomically intact in the retinal explants we used, and thus could contribute to the circadian light responses we recorded. In this regard, it is interesting to note that dopaminergic amacrine neurons are light responsive [72] and that a subpopulation of them exhibit sustained light responses driven by melanopsin-expressing intrinsically photoreceptive ganglion cells in a novel intraretinal photic pathway [73]. Thus, melanopsin ganglion cells may contribute to the entrainment of both retinal and SCN neural oscillators.

Based on our results, we propose that endogenous retinal dopamine serves to synchronize the inner retinal circadian clock to the external light/dark cycle. In vivo, retinal dopamine release is rhythmic, exhibiting a peak near dawn each day, due to both circadian and light-driven regulation of retinal dopamine [74]. Light increases the activity of retinal tyrosine hydroxylase, the key enzyme in dopamine synthesis, within 15 min of light onset [75], and evokes a sharp burst of dopamine synthesis and utilization within 30 min of light onset [76]. On a molecular level, dopamine mediates acute light induction of the *Per1* gene in the mouse retina [68] and resets the *Xenopus* photoreceptor clock through induction of *xPer2* [70,71]. The phase dependence of dopamine-induced phase shifts we have observed corresponds with the likely phase-resetting response to light, with phase advances induced in the early subjective day and phase delays in the early subjective night. Phase delays induced by light pulses in the early subjective night were significantly reduced by blockade of dopamine D1 receptors. The circuitry of mammalian retinal dopaminergic amacrine cells [72,73], the molecular action of dopamine on retinal clock genes [68], and our direct demonstration of dopamine effects on clock phase and light resetting of retinal clock phase, all indicate that dopamine transmission likely mediates light entrainment of the inner retinal mammalian circadian clock through its action on *Per* gene levels and rhythms. Importantly, light-induced phase shifts were not completely blocked by D1 antagonists and dopamine agonists could not mimic the full amplitude of light-induced phase shifts, suggesting that other neurotransmitters are also involved in the light entrainment process.

In addition to setting the phase of the retinal clock, dopamine is a key output of the retinal clock, inducing many of the functional changes in retinal neurons and circuits that define the “day” state of retinal function, such as cone-dominated processing in visual circuits and ERG amplitude [13,66,77], and mediating circadian rhythms in behaviorally measured visual sensitivity [78]. Thus, retinal dopamine apparently plays dual roles in the circadian organization of the retina, serving as an output signal that mediates many of the physiological, morphological, and trophic rhythms in the retina, as well as an input signal to regulate the phase of the molecular clock mechanism in relation to the external light/dark cycle. As both a key input to and output from the mammalian retinal clock, dopamine plays a central role in retinal circadian organization and is an important point for mechanistic intervention in ocular processes and pathologies regulated by the circadian clock.

### GABA and Retinal PER2::LUC Rhythms

In the current study, we found that GABA_A_ and GABA_C_ receptor blockade significantly increased the amplitude of retinal PER2::LUC rhythms, whereas application of exogenous GABA damped rhythmic amplitude, and with prolonged application, stopped the retinal circadian clock. The high concentrations of exogenous GABA (>100 μM) necessary to modulate retinal PER2::LUC rhythms in these experiments are likely due to uptake by high-affinity GABA transporters present on a range of retinal neurons, as well as on retinal Müller cells [79]. Previous electrophysiological experiments have demonstrated, for example, that application of 100 μM GABA produces near-maximal effects on isolated retinal ganglion cells, but higher concentrations are necessary to produce detectable effects in retinal slice preparations [80]. Our finding that the effects of GABA can be mimicked or blocked by GABA_A_ and GABA_C_ receptor agonists and antagonists indicates that the effects of GABA on retinal PER2::LUC rhythms are receptor mediated and specific.

Our data establish that GABAergic regulation is a common mechanism across neural circadian clocks because blockade of GABA receptors in SCN explants also increases *Per1::luc* rhythmic amplitude [65], and application of GABA resets the phase of spike frequency rhythms in isolated SCN neurons [81]. However, the ability of exogenous GABA to suppress substantially PER2::LUC rhythms is unique to the retina, as similar experiments with SCN slices do not yield similar results. The increased effectiveness of GABA as a modulator of retinal PER2 expression is likely due to the specific retinal expression of the GABA_C_ receptor, which engenders nondesensitizing responses and prolongs greatly the effectiveness of any GABA stimulation [82–84].

The inhibitory action of GABA on the amplitude of retinal PER2::LUC rhythms could act through damping of circadian rhythms in individual retinal neurons, or through desynchronizing individual oscillators, and differentiating these mechanisms will require single-cell resolution studies of retinal circadian rhythms. A functional consequence of reducing rhythmic amplitude, predicted by limit cycle models of circadian oscillators, is to increase sensitivity to phase-resetting stimuli. Thus, a potential role of GABA in neural circadian oscillators is to enhance the effectiveness of endogenous phase-communicating substances, such as dopamine in the retina [70], or vasoactive intestinal polypeptide (VIP) and gastrin-releasing peptide (GRP) in the SCN [85,86], in resetting the rhythms of neuronal oscillators by limiting the amplitude of the intracellular molecular clock oscillations.

On a mechanistic level, our data suggest that GABA acts to suppress the amplitude of retinal PER2::LUC rhythms through membrane hyperpolarization and casein kinase activation. The mechanism by which GABA may stimulate retinal casein kinases is unknown at this point. CKI has been shown to physically associate with GABA_A_ receptors in a rhythmic manner in the SCN [58], and CKII has been shown to phosphorylate GABA_C_ receptor subunits [59]. These types of physical associations between GABA receptors and casein kinases have been assumed to be for receptor modulation, but could provide a link between persistent activation of GABA receptors and activation of casein kinases as well. It is noteworthy that the circadian association between CKIɛ–CKIδ and GABA_A_ receptors in the rat SCN peaks at CT 6–7 [58], when GABA content in the SCN is high [87] and PER levels are low [88,89], and then drops to low levels in early subjective night when PER levels peak. This may imply a novel clock regulatory mechanism in which PER and GABA receptors compete for association with casein kinases. It will be of importance to test this hypothesis in the mouse retina, where GABA exhibits significant effect on PER2::LUC rhythms. Alternatively, casein kinases are regulated by protein phosphatases and by metabotropic glutamate receptors [90,91], and perhaps persistent GABA stimulation alters these pathways. It will be of interest in the future to elucidate more fully the mechanistic relationship of GABA to casein kinase and its effect on the retinal clock.

### GABA and Retinal Circadian Clock Organization

Our finding that GABA plays key roles in the retinal circadian clock draws attention to GABAergic neurons as sources of these retinal circadian signaling molecules and potential sites of rhythms generation. The full complement of core clock genes is expressed in GABAergic horizontal cells and dopaminergic amacrine cells, which also express GABA [43]. In addition, transcription of the *Per1* clock gene has been shown to occur in most GABAergic amacrine cells, and rhythmically in the dopaminergic and nitric-oxide synthase (NOS)-positive subtypes of GABAergic amacrine cells [42,92]. Thus, there is a strong correlation of core clock gene expression within GABAergic retinal neurons, suggesting a molecular basis for circadian regulation of this neurotransmitter.

Retinal GABA turnover rate and release show rhythmic variations under constant darkness condition, with their levels higher in the subjective night [21]. Furthermore, GABA increases melatonin content in a dose-dependent manner in the hamster retina [93]. Our results suggest that GABA could, through membrane hyperpolarization and activation of casein kinase, stimulate the degradation of accumulated PER proteins on the falling phase of their rhythm (which roughly corresponds to the night state), acting to remove feedback inhibition on *Per* transcription and thereby preparing retinal oscillators for transcriptional activation that characterizes the “day” state. Importantly, GABA is not acting merely through suppression of dopamine release, as dopamine depletion does not mimic the effects of GABA. Taken together, these data are consistent with the notion that one role of GABA in the retinal circadian clock is to function as an analog for darkness and enforce the “night” state in the mammalian retina, along with melatonin.

Our finding that the GABA_C_ receptor is a critical co-mediator of GABA influence on retinal PER2::LUC rhythms suggest roles for neurons in the outer half of the retina in circadian clock function. The GABA_C_ receptor has a restricted distribution in the mammalian retina, with the primary site of expression on bipolar cell synaptic terminals in the inner plexiform layer where it mediates recurrent feedback from GABAergic amacrine cells [94], and a secondary site of expression in mouse cone photoreceptors [95]. Thus, the balance of results suggests that retinal bipolar cells, some of which coordinately express all the core clock genes [43], and perhaps photoreceptors, are important targets of GABAergic regulation in the retinal circadian clock network. Convincing evidence for the presence of GABA_C_ receptors on rod photoreceptors and horizontal, amacrine, and ganglion cells in the mammalian retina is still lacking; however, GABA_A_ receptors may mediate the action of GABA on these cell types, because the GABA_A_ receptor agonist muscimol alone partially reduced rhythmic PER2::LUC amplitudes (Figure S3A), and GABA_A_ receptors have been detected in these retinal cell types [31].

### Model for the Mouse Retinal Circadian Clock Organization

**Figure 11:**
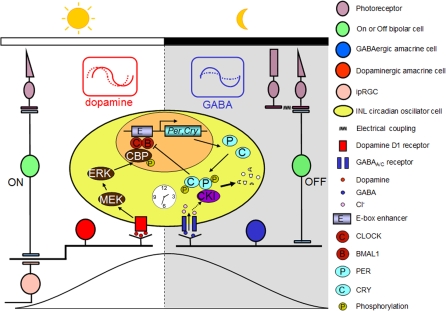
Model for the Circadian Clock Organization in the Mouse Retina Dopaminergic amacrine cells (red) and GABAergic cells (blue) reinforce the autonomously generated “day” and “night” states of oscillator cells (yellow) in the inner nuclear layer (INL) through rhythmic secretion of dopamine and GABA, respectively. Dopamine transmission stimulated by excitatory light input from M-cones via ON bipolar cells and/or from intrinsically photosensitive retinal ganglion cells (ipRGCs) likely acts to phase-shift retinal circadian oscillators and reinforce the rising phase of the “day” state of the retinal clock. At night (i.e., molecularly, roughly the falling phase of PER protein rhythms), excitatory input from OFF bipolar cells enhances the activity of GABA, which suppresses rhythmic amplitude of PER oscillations through the GABA_A_ and GABA_C_ receptors and facilitates the molecular resetting effects of dopamine. In addition, endogenous GABA is proposed to reinforce the falling phase of PER rhythms through fostering degradation of accumulated PER protein.

In summary, the present data are consistent with the hypothetical model shown in Figure 11 for circadian organization of the mammalian retina in which GABAergic and dopaminergic neurons play key roles through rhythmic secretion of dopamine and GABA, and the subsequent molecular actions of dopamine and GABA on PERs, via transcriptional activation and degradation. In this model, dopamine, which exhibits both circadian and light-induced elevation in the day phase [19,75], reinforces the rising phase of *Per* molecular rhythms during the day through up-regulation of *Per* genes via the mitogen-activated extracellular signal-regulated kinase (ERK) kinase (MEK) and the cAMP-responsive element-binding protein (CREB) binding protein (CBP) [68]. In terms of phase resetting, increasing dopaminergic stimulation on the initial rising phase of the PER2 rhythms, corresponding to the early day, resulted in phase advances, whereas increasing dopaminergic stimulation near the peak in PER2 expression, corresponding to the early night, resulted in phase delays. We propose that light stimulation of dopaminergic neurons through M-cones and ON bipolar cells, as well as intrinsically photosensitive retinal ganglion cells (ipRGCs) [73] at these phases would act to synchronize molecular retinal circadian rhythms to the light/dark cycle. In contrast, retinal GABA exhibits elevated turnover and release at night [21], perhaps from excitatory input from OFF bipolar cells to GABAergic amacrine cells, and in this model, stimulates the degradation of accumulated PER proteins through casein kinase activation, acting to remove feedback inhibition on *Per* transcription, and thereby preparing retinal oscillators for transcriptional activation in the next “day” state. GABA might also facilitate the molecular resetting effects of dopamine through negatively regulating the amplitude of retinal PER rhythms. Thus, GABA transmission acts to reinforce the “night” state of the retinal clock. The idea that GABA serves as a “dark” signal is consistent with previous findings that GABA and the GABA_A_ receptor agonist muscimol cause dark-adaptive retinomotor movements in the *Xenopus* and fish retinas [96,97] and that GABA suppresses tyrosine hydroxylase activity [98] and inhibits dopamine release [49].

Future studies employing our in vitro retinal explant culture system and other strategies can test our working hypothesis regarding the organization of the mammalian retinal circadian clock, and further elucidate the cellular and molecular mechanisms of retinal circadian clocks and their influence on visual function. The highly ordered and well-characterized property of retinal circuitry will facilitate elucidation of the general principles of the circadian pacemaking system.

## Materials and Methods

### Animals.

*mPer2^Luc^* knockin mice, which initially were on a 129SvEv X C57BL/6J genetic background [44], were maintained as a continuous backcross to C57BL/6J for 13 generations. The resulting *mPer2^Luc^* mice were crossed with C3H *rd1* mice (The Jackson Laboratory) to produce *mPer2^Luc^* mice that are heterozygous for the *rd1* gene mutation and are genetically capable of producing melatonin. Unless otherwise specified, the mice used in the experiments were B6C3F1 *mPer2^Luc^* mice. All animal use was conducted in accordance with the guidelines of the Vanderbilt University Animal Care Division and the Association for Research in Vision and Ophthalmology (ARVO) Statement for the Use of Animals.

### Retinal explant culture.

*mPer2^Luc^* mice 28–60 d of age were killed at approximately ZT 4 during the day phase (4 h after lights on in the mouse colony). Eyes were enucleated and placed in Hank's balanced salt solution (HBSS; Invitrogen). Retinas were isolated and cut into two pieces. Each piece of retina was placed with the ganglion cell layer up on Millicell culture membrane (Millipore) and gently flattened with two end-blunted glass pipettes. The membrane was first transferred to 1.0 ml of neurobasal medium (Invitrogen) supplemented with 2 mM l-glutamine (Sigma-Aldrich), 2% B27 (Invitrogen), 25 units/ml penicillin, and 25 μg/ml streptomycin (Invitrogen), incubated in 5% CO_2_ incubator at 37 °C for 24 h, and then transferred to 1.0 ml of medium 199 (Sigma-Aldrich) supplemented with 0.7 mM l-glutamine, 4 mM sodium bicarbonate (Sigma-Aldrich), 10 mM Hepes (Sigma-Aldrich), 20 mM d-glucose (Sigma-Aldrich), 2% B27, 0.1 mM beetle luciferin (Promega), 25 units/ml penicillin, and 25 μg/ml streptomycin. Bioluminescence was measured with a LumiCycle (Actimetrics). Cultures were maintained at 37 °C. LumiCylce (Actimetrics) software was used to calculate peak-to-trough amplitude, and ClockLab software (Actimetrics) was used to determine the peak time (maximum phase) of PER2::LUC rhythms from the raw data.

### SCN explant culture.

At approximately ZT 4, *mPer2^Luc^* knockin mice were killed by cervical dislocation, the brains rapidly extracted following decapitation and immediately placed in cold HBSS. Coronal brain sections (250-μm thickness) were cut with a Vibratome (World Precision Instruments), transferred to cold HBSS, and sorted under a dissecting microscope. Slices containing the SCN were trimmed to rectangles 3 × 3 mm with a pair of scalpels, and then cultured on Millicell culture membranes with 1.0 ml of DMEM (Sigma), supplemented with 4 mM sodium bicarbonate, 10 mM Hepes, 20 mM d-glucose, 2% B27, 0.1 mM beetle luciferin, 25 units/ml penicillin, and 25 μg/ml streptomycin. Bioluminescence was continuously measured with a LumiCycle. Cultures were maintained at 37 °C.

### Vertical retinal slice imaging.

Freshly isolated retinas were attached tightly to Millipore membrane filters, with the ganglion cell layer facing the filters, sliced into 150-μm-thick sections with a tissue slicer (Stoelting), transferred onto Millicell membrane inserts, and cultured with 1.0 ml of neurobasal medium, and then incubated in 5% CO_2_ incubator at 37 °C for 1–2 d. Immediately before imaging, 0.1 mM beetle luciferin was added to the culture medium. Images of 30-min exposure duration were collected using a 20× objective (NA 0.35) coupled directly to a PIXIS camera system (Princeton Instruments) cooled to −70 °C. Signal-to-noise ratio was improved by 2 × 2 binning of pixels. Data were analyzed with Metamorph software (Molecular Devices).

### Immunocytochemistry.

Retinal explant whole mounts were fixed in phosphate-buffered saline (PBS) containing 4% paraformaldehyde (PFA), blocked with 1% bovine serum albumin (BSA) and 0.3% Triton X-100 in 0.1 M PBS for 2 h, and then incubated in the primary antibodies at 4 °C for 48 h. The primary antibodies used were: 1:500 sheep anti-tyrosine hydroxylase (Chemicon); 1:1,000 rabbit anti-cone arrestin antibody Luminaire junior; 1:1,000 rabbit anti-M-opsin; 1:1,000 rabbit anti-S-opsin (cone antibodies were kind gifts from Dr. Craft, University of Southern California); 1:1,000 mouse anti-calbindin (Chemicon); 1:1,500 rabbit anti-CaBP5 (a gift from Dr. Haeseleer, University of Washington); 1:3,000 mouse anti-calretinin (Chemicon); and 1:2,000 rabbit anti-melanopsin (a gift from Dr. Provencio, University of Virginia). After rinsing, a secondary incubation was performed for 2 h with either 1:500 Alexa Fluor 488 donkey anti-sheep IgG, 1:500 Alexa Fluor 488 donkey anti-rabbit IgG, or 1:500 Alexa Fluor 594 donkey anti-mouse IgG (all purchased from Molecular Probes). Samples were mounted and cover slipped using Vectashield (Vector Laboratories) and then visualized using Zeiss LSM5 PASCAL confocal microscopy (Carl Zeiss).

### HPLC.

The total content of dopamine from retinal explants was determined by HPLC in the Vanderbilt Neurochemistry Core Lab. The HPLC system consisted of a 515 HPLC pump (Waters), a 717 plus autosampler (Waters), a Decade II (oxidation: 0.5) electrochemical detector (Antec Leyden), and a Nucleosil (5 μm, 100 Å) C18 HPLC column (150 × 4.6 mm; Phenomenex). The retinal samples were first homogenized in 200 μl of 0.1 M TCA, which contains 10 mM sodium acetate, 0.1 mM EDTA, and 10.5% methanol (pH 3.8), and then centrifuged at 10,000 *g* for 20 min, filtered on a 0.2-μm pore size filter. A total of 20 μl of each sample was directly injected into the HPLC system. The mobile phase was composed of 89.5% 0.1 M TCA, 10 mM sodium acetate, 0.1 mM EDTA, and 10.5% methanol (pH 3.8). The mobile phase flow rate was set to 0.6 ml/min and the electrochemical detector to 33 °C. HPLC control and data acquisition were managed by Millennium 32 software (Waters).

### Quantitative real-time PCR.

To determine the transcript levels of core clock genes in GABA- and vehicle-treated retinal explants, retinal explants were homogenized 8 h after treatment started at the beginning of the third circadian cycle, and total RNA extraction and real-time RT-PCR were performed as previously described [43]. The housekeeping gene glyceraldehyde-3-phosphate dehydrogenase (GAPDH) was used as a reference gene for normalization. For data analysis, the threshold cycle (*C*t) was converted to absolute amount of transcript (*E^−C^* ^t^) and presented as *E^ C^* ^t GAPDH -*C*t gene of interest^. The mean value of *E^ C^* ^t GAPDH -*C*t gene of interest^ for vehicle was normalized to 1.0. A Student's *t*-test was applied to compare clock gene expression levels between treatment and vehicle.

### Data analysis.

Student's *t*-test was routinely performed to compare the difference in the damping rate, period, phase, and A_4_/A_2_ ratio between drug- and vehicle-treated samples. For GABA_A_ and GABA_C_ antagonists treatment, two-way repeated-measures ANOVAs were run for both drug and vehicle control in which the variances were homogeneous, as indicated by a significant Levene test. For post hoc analysis of significant interactions, independent *t*-tests with a Bonferroni-corrected alpha (the alpha was Bonferroni corrected by the number of comparisons) were used to compare the effects of drug for each cycle.

## Supporting Information

Figure S1 Mouse Retinas Show Low Amplitude PER2::LUC Oscillations When Directly Cultured in Medium 199 (A) or DMEM (B) (1.61 MB TIF)

Figure S2 Temporal Expression Profiles of Six Core Clock Genes in the Mouse Retina under 12-h Light:12-h Dark (Filled Circles) and Constant Darkness (Open Circles) Conditions Quantitative real-time RT-PCR and data analysis were performed as in [43]. Data are presented as means ± SEM from five B6C3F1 mice and expressed as a percentage of the maximum mean expression. Points at ZT/CT [2] are duplicates of ZT/CT 2 replotted to show 24-h trends. Under 12-h light:12-h dark condition, one-way ANOVA revealed *p* < 0.001 for *Per2*, *Cry2*, and *Bmal1*, and *p* < 0.05 for *Per1*, *Cry1*, and *Clock*. The peak-to-trough amplitudes for *Per1*, *Per2*, *Cry1*, *Cry2*, *Clock*, and *Bmal1* expression were 1.27-, 1.50-, 1.29-, 1.49-, 1.33-, and 1.30-fold, respectively. Under constant darkness condition, one-way ANOVA revealed *p* < 0.001 for *Per1*, *Per2*, and *Cry1*, *p* < 0.05 for *Bmal1*, and *p* > 0.05 for *Cry2* and *Clock*. The peak-to-trough amplitudes for *Per1*, *Per2*, *Cry1*, and *Bmal1* expression under constant darkness were 1.40-, 2.12-, 1.47-, and 1.20-fold, respectively.
(4.44 MB TIF)

Figure S3 All the Major Retinal Cell Types Are Morphologically Intact in Cultured Mouse Retinal Explants Flat-mount view showing immunoreactivity for cone photoreceptors ([A–C]; anti-cone arrestin, M-opsin, and S-opsin; green), horizontal cells ([D]; anti-calbindin; focused on the horizontal cell layer; red), bipolar cells ([E]; anti-CaBP5; green), amacrine cells ([F]; anti-calretinin; red), and melanopsin-containing ganglion cells ([G]; anti-melanopsin; green). Retinal explants were cultured for 5 d in vitro (DIV; 1 d in 5% CO_2_ incubator and 4 d in the LumiCycle) before immunocytochemistry. Scale bar represents 20 μm.
(3.55 MB TIF)

Figure S4 GABA Does Not Suppress SCN Ensemble PER2::LUC Rhythms (4.21 MB TIF)

Figure S5 Actions of GABA Receptor Agonists on Retinal PER2::LUC Rhythms Shown are representative PER2::LUC bioluminescence traces of mouse retinal explants treated with: (A) the GABA_A_ receptor agonist muscimol (200 μM); (B) the GABA_B_ receptor agonist baclofen (200 μM); (C) the GABA_C_ receptor agonist CACA (50 μM); (D) muscimol (200 μM) and baclofen (200 μM); (E) baclofen (200 μM) and CACA (50 μM); and (F) muscimol (200 μM) along with baclofen (200 μM) and CACA (50 μM). Bars indicate the duration of treatment.
(4.19 MB TIF)

Figure S6 Actions of GABA Receptor Antagonists on the Inhibitory Effect of GABA on Retinal PER2::LUC Rhythms Shown are representative PER2::LUC bioluminescence traces of mouse retinal explants treated with 1 mM GABA along with: (A) the GABA_A_ receptor antagonist SR-95531 (40 μM); (B) the GABA_B_ receptor antagonist CGP-35348 (100 μM); (C) the GABA_C_ antagonist TPMPA (100 μM); (D) SR-95531 (40 μM) and CGP-35348 (100 μM); (E) CGP-35348 (100 μM) and TPMPA (100 μM); and (F) SR-95531 (40 μM), CGP-35348 (100 μM), and TPMPA (100 μM). Bars indicate the duration of treatment.
(4.17 MB TIF)

Figure S7 Actions of GABA Receptor Antagonists on Retinal PER2::LUC Rhythms Shown are representative PER2::LUC bioluminescence traces of mouse retinal explants treated with: (A) the GABA_A_ receptor antagonist SR-95531 (40 μM); (B) the GABA_B_ receptor antagonist CGP-35348 (100 μM); (C) the GABA_C_ antagonist TPMPA (100 μM); (D) SR-95531 (40 μM) and CGP-35348 (100 μM); and (E) CGP-35348 (100 μM) and TPMPA (100 μM). Bars indicate the duration of treatment.
(4.24 MB TIF)

## Abbreviations

ACh: acetylcholine
CKI: casein kinase I
Cry: Cryptochrome
Ct: threshold cycle
CT: circadian time
cx: connexin
GABA: gamma-aminobutyric acid
GAPDH: glyceraldehyde-3-phosphate dehydrogenase
HPLC: high-performance liquid chromatography
L-AA: l-ascorbic acid
L-AMPT: α-methyl-l-tyrosine
*Per,*: *Period;*
PER2::LUC: PERIOD2::LUCIFERASE
RT-PCR: reverse-transcriptase PCR
SCN: suprachiasmatic nucleus
SEM: standard error of the mean
TTX: tetrodotoxin
ZT: Zeitgeber time
